# Hospitals and General Practitioners

**Published:** 1915-07-03

**Authors:** 


### The Hospital

The Workers' Newspaper of Administrative Medicine and Institutional Life, Administration, National Insurance and Health.

No. 1516, Vol. LVIII. SATURDAY, JULY 3, 1915. PRICE ONE PENNY

### HOSPITALS AND GENERAL PRACTITIONERS.

The present strained, or even critical, condition of the medical and hospital world has its obvious anxieties, and all concerned feel the disadvantages which arise from it. Boards of governors are concerned about increased expenses of administration and diminished incomes; medical staffs are overburdened with enlarged responsibilities and duties; and patients from civil life find the accommodation nominally provided for them limited by the necessities of sick and wounded soldiers. The resident staff, it is true, has the benefit of an increased salary, though even here there is usually an added burden of work; and concerning the younger men, at all events, there is the painful question whether their real duty does not lie elsewhere. All this makes a somewhat unhappy picture, and no immediate relief from the strain can be anticipated. The penalties of war fall on all of us in greater or less measure, and everyone in his own sphere must listen to the patriotic claim which demands both the cheerful acceptance of inevitable inconveniences and a readiness to make the best of circumstances. Not all may stand in the limelight, but everyone may put his shoulder to the wheel. If with any of us it has to be admitted that life and work are "as usual," it is high time for a reconsideration of the position. When the strain will cease not even the prophets can tell, but it is certain that sustained and organised effort is necessary if our great voluntary hospital system is to be carried safely through the crisis, and carried through it must be if the interests of the sick poor and of medical efficiency are to be maintained.

There are certain influences arising from the war which may possibly have a beneficial action on the position and fate of our hospitals. One is an increased attention to the work and wants of these institutions, due to the large and essential part they are playing in the care of men who have suffered in the defence of the national cause. It is unthinkable that this service can be readily forgotten, and the recognition of it ought to mean an enlarged area of practical sympathy for the work which the hospitals undertake in time of peace. Men, and women too, fall and suffer in discharging the duties of civil life, and many of them can have no effective succour save in the hospitals. The patriotism which desires an effective national life is not, or ought not to be, limited to times of war and crisis. In such a life the hospitals play an essential part, and they vindicate their claim at all dates and before all classes. Just now they appear prominently in the public eye, and from this much may be hoped when once more the country enjoys the blessings of peace.

Another possible good which may come to the hospitals and to the medical profession from the present crisis is a closer association between the hospitals and those who practise in their neighbourhood. Just now—and the demand will probably be an increasing one—general practitioners are being called on to give help to the hospitals in their difficulties. Resident medical officers are a short supply, and the duties which in normal circumstances fall upon them may be, at least in part, undertaken by those engaged in private practice in the vicinity of the hospitals. In this way there is taking place a much closer association between the hospital staffs and the outside practitioners than usually exists. Is it too much to hope that such association will be continued and even extended? The benefit, we believe, would be in both directions, and all concerned ought to try to secure it.

Unfortunately, it is to be feared, the relations here considered are not always of the most pleasant and helpful order; and probably for this state of matters there are responsibilities on both sides. Misunderstandings readily arise in medical work, and most of them are due to some failure in frankness or in courtesy. It is in this fashion that there is often developed towards the hospital a feeling of hostility and suspicion, whereas quite plainly there ought to be an atmosphere of co-operation and of mutual help. What is to be recognised is that the claims of professional consideration and courtesy apply as truly to hospital practice as to private practice, and if an improved state of matters is to be established these claims must receive attention. Only too often, unhappily, they are largely or completely ignored. It is not much to ask that when a patient, who has obviously been under the care of a private practitioner, is admitted to hospital the fact shall be intimated to the practitioner concerned. He ought to receive an intimation of the date and hour when the patient will be examined by the physician, or when an operation, if this is necessary, will be performed. In this way his position is duly recognised and he has the opportunity of following the clinical developments of his case. Nor is the advantage solely to the private practitioner. On the contrary, it is from him, and often from him only, that a true knowledge of the earlier stages of the history can be obtained, and this knowledge not infrequently is essential to a sound understanding of the question at issue. The advantages of consultation are recognised in private practice, and they apply with equal force to the hospital clinic and to the hospital patient. There will be occasions when from urgency of the circumstances or other causes the practice here advocated is impossible. But it ought to be kept in mind as the course which it is desirable to follow, and even though an ideal is unattainable in every instance, it is always a stimulus to effort. On the other hand, it is fair to say that the outside practitioner must not expect impossibilities, and must be ready to apply a reasonable charity to actions which may seem at times to ignore his position and responsibility. If he is willing to be helpful he will hardly fail to command an answering recognition. From such a sense of mutual goodwill the hospital would grow to be a centre where difficulties would be discussed and clinical knowledge extended. It would, in short, be a kind of post-graduation school in its own individual district, and all concerned would feel the benefit. In existing circumstances the pressure is too great to admit of anything precise and co-ordinated, but men who were previously largely separated, if not antagonistic, are meeting together on common ground and with a common interest, and from this it is reasonable to expect that improved relations will be established. What is further to be desired is that from such association some permanent advantages in the shape of mutual helpfulness and co-operation will be established.

